# Supplementary material for: Ethnic disparities in hospitalisation and hospital-outcomes during the second wave of COVID-19 infection in east London
Source: Sci Rep. 2022 Mar 8;12:3721. doi: 10.1038/s41598-022-07532-6 (PMC8904852; doi:10.1038/s41598-022-07532-6)

Supplementary material

Definition of key variables

Ethnicity
We defined ethnic groups using the 16+1 categories defined in the 2001 census which form the UK national mandatory standard for the collection and analysis of ethnicity in the NHS data dictionary. Importantly, in the UK 'Asian' ethnic category refers predominantly to those of a South Asian background (including Indian, Pakistani and Bangladeshi), while patients of a Chinese background are placed in the 'Other Ethnic Groups' category.

White	A British<br>B Irish<br>C Any other White background
Mixed	D White and Black Caribbean<br>E White and Black African<br>F White and Asian<br>G Any other mixed background
Asian or Asian British	H Indian<br>J Pakistani<br>K Bangladeshi<br>L Any other Asian background
Black or Black British	M Caribbean<br>N African<br>P Any other Black background
Other Ethnic Groups	R Chinese<br>S Any other ethnic group
+1 category	Z Not stated (Reserved for cases where patients declined to provide information)

In order to preserve statistical power to detect differences between groups, pre-specified analysis was carried out between ethnicity defined by the 5-high level groups White, Mixed, Asian or Asian British, Black or Black British and Other with merging of the "Mixed" and "Other" categories. Category Z was excluded from our primary analysis as were cases where no ethnicity data was recorded (Unknown).

Index of Multiple Deprivation
Index of Multiple Deprivation (IMD) was defined from patient home address postcode using UK government statistics (https://www.gov.uk/government/statistics/english-indices-of-deprivation-2019). Matching of Lower-layer Super Output Areas (LSOAs) was undertaken against the Office of National Statistics Postcode Directory (ONSPD) February 2020 datafile (https://geoportal.statistics.gov.uk/datasets/ons-postcode-directory-february-2020: accessed on 1st May 2020). IMD was presented as quintiles within England using raw scores for descriptive results and quintiles within the study cohort in multivariable analysis.

Smoking
History of tobacco use was defined by presence of the WHO ICD-10 codes F17·1-F17·2, Z72·0, Z87·8, Z71·6 and T65·2.

Comorbidity
Diagnosis of co-morbidities and assignment of Charlson Comorbidity Index was based on mapping from ICD-10 coding from previous admissions using the mapping of Quan H, et al.
Quan H, et al. Coding algorithms for defining comorbidities in ICD-9-CM and ICD-10 administrative data. Med Care 2005;43(11):1130-9.
Diagnosis of Hypertension was based on mapping ICD-10 codes to the Elixhauser comorbidity index.
Elixhauser A, et al. Comorbidity measures for use with administrative data. Med Care 1998;36:8-27.

Hospital frailty risk score
Hospital frailty risk score was calculated from mapping ICD-10 coding of hospital attendances.
Gilbert T, et al. Development and validation of a Hospital Frailty Risk Score focusing on older people in acute care settings using electronic hospital records: an observational study. Lancet 2018;391(10132):1775-1782.

Acute Kidney injury
Acute kidney injury (AKI) within first 7 days of admission was defined using the KDIGO 2012 creatinine criteria either a 1·5-fold rise over baseline within 7 days or 26 μmol rise within 48 hours. Baseline creatinine will be the median value in the 7 to 365 days before hospitalisation. Absent baseline creatinine was determined based on an eGFR of 75 ml/min/1·72m^2 using the CKDepi formula or the admission value whichever was lower.

Chronic kidney disease
History of chronic kidney disease (CKD) using baseline eGFR was calculated using last creatinine value available from results earlier than 7 days before hospitalisation. CKD was defined as baseline eGFR below 60 ml/min/1·72m^2.

Table S1: IMD breakdown by National IMD deciles showing the majority of our study population residing in IMD deciles 1-4.

Characteristic	n	White, n = 1,805[1]	Black, n = 634[1]	Asian, n = 1,983[1]	Mixed/Other, n = 433[1]	Unknown, n = 678[1]	p-value[2]
National IMD Decile	5,490						<0.001
1		43 (2.4%)	29 (4.6%)	33 (1.7%)	14 (3.3%)	21 (3.1%)	
2		368 (21%)	206 (33%)	506 (26%)	118 (28%)	170 (25%)	
3		468 (26%)	182 (29%)	705 (36%)	125 (29%)	210 (31%)	
4		261 (15%)	107 (17%)	378 (19%)	74 (17%)	117 (17%)	
5		154 (8.6%)	56 (8.9%)	136 (6.9%)	41 (9.6%)	55 (8.2%)	
6		155 (8.7%)	26 (4.1%)	84 (4.3%)	23 (5.4%)	37 (5.5%)	
7		98 (5.5%)	8 (1.3%)	47 (2.4%)	12 (2.8%)	21 (3.1%)	
8		104 (5.8%)	6 (1.0%)	36 (1.8%)	11 (2.6%)	20 (3.0%)	
9		85 (4.7%)	7 (1.1%)	32 (1.6%)	6 (1.4%)	13 (1.9%)	
10		55 (3.1%)	2 (0.3%)	16 (0.8%)	3 (0.7%)	6 (0.9%)	
Unknown		14	5	10	6	8	

[1]n (%)
[2]Pearson's Chi-squared test

Table S2: Rockwood (clinical) and Hospital Frailty Risk (coding-based) scoring within our study cohort.

Characteristic	n	White, n = 1,805[1]	Black, n = 634[1]	Asian, n = 1,983[1]	Mixed/Other, n = 433[1]	Unknown, n = 678[1]	p-value[2]
Rockwood	2,435						0.002
1. Very fit		23 (2.2%)	6 (2.2%)	20 (2.6%)	4 (2.5%)	7 (3.4%)	
2. Well		90 (8.7%)	27 (10%)	80 (11%)	29 (18%)	28 (13%)	
3. Managing well		234 (23%)	77 (29%)	216 (28%)	52 (32%)	49 (24%)	
4. Vulnerable		191 (18%)	56 (21%)	164 (22%)	26 (16%)	43 (21%)	
5. Mildly frail		149 (14%)	33 (12%)	89 (12%)	10 (6.1%)	27 (13%)	
6. Moderately frail		199 (19%)	39 (14%)	114 (15%)	26 (16%)	28 (13%)	
7. Severely frail		113 (11%)	23 (8.6%)	60 (7.9%)	11 (6.7%)	20 (9.6%)	
8. Very severely frail		38 (3.7%)	8 (3.0%)	13 (1.7%)	5 (3.1%)	5 (2.4%)	
9. Terminally ill		0 (0%)	0 (0%)	2 (0.3%)	0 (0%)	1 (0.5%)	
Unknown		768	365	1,225	270	470	
HFRS	5,518						<0.001
<5		964 (53%)	377 (60%)	1,326 (67%)	315 (73%)	506 (75%)	
5-15		627 (35%)	180 (29%)	509 (26%)	98 (23%)	147 (22%)	
>15		211 (12%)	74 (12%)	144 (7.3%)	19 (4.4%)	21 (3.1%)	
Score (median)		4.3 (1.0, 9.9)	3.6 (0.5, 8.4)	2.3 (0.0, 6.5)	1.8 (0.0, 5.3)	1.5 (0.0, 4.9)	<0.001
Unknown		3	3	4	1	4	

[1]n (%); Median (IQR)
[2]Pearson's Chi-squared test; Kruskal-Wallis rank sum test

Table S3: Age and ethnicity breakdown of First Wave COVID-19 associated hospital admission population residing within Tower Hamlets, Newham or Waltham Forest.

First Wave n = 1409	White	Black	Asian	Mixed	Other	Unknown
Age 16 to 19	2	0	6	0	0	1
Age 20 to 24	3	3	6	1	0	2
Age 25 to 29	5	3	24	0	3	3
Age 30 to 34	7	6	25	1	2	6
Age 35 to 39	12	8	20	0	8	9
Age 40 to 44	12	13	32	0	2	7
Age 45 to 49	19	15	44	1	11	7
Age 50 to 54	31	23	34	1	8	16
Age 55 to 59	40	32	35	1	18	17
Age 60 to 64	40	24	47	1	7	11
Age 65 to 69	27	19	48	1	8	9
Age 70 to 74	50	26	22	1	10	16
Age 75 to 79	51	28	22	0	5	10
Age 80 to 84	65	39	38	1	3	8
Age 85 and over	109	33	28	2	7	8
Total	473	272	431	11	92	130
% Total	33.6%	19.3%	30.6%	0.8%	6.5%	9.2%

Table S4: Age and ethnicity Breakdown of Second Wave COVID-19 associated hospital admission population residing within Tower Hamlets, Newham or Waltham Forest.

Second Wave n = 3891	White	Black	Asian	Mixed	Other	Unknown
Age 16 to 19	8	6	16	0	1	2
Age 20 to 24	17	7	38	5	8	16
Age 25 to 29	26	13	81	1	6	21
Age 30 to 34	44	21	107	2	13	31
Age 35 to 39	44	23	116	2	18	29
Age 40 to 44	50	24	130	1	16	39
Age 45 to 49	51	34	137	1	25	38
Age 50 to 54	67	39	119	4	25	36
Age 55 to 59	79	59	142	3	24	34
Age 60 to 64	92	48	173	6	34	35
Age 65 to 69	85	39	166	1	27	23
Age 70 to 74	125	40	106	1	16	21
Age 75 to 79	115	47	95	1	11	17
Age 80 to 84	105	52	130	3	13	20
Age 85 and over	178	33	98	1	13	22
Total	1086	485	1654	32	250	384
% Total	27.9%	12.5%	42.5%	0.8%	6.4%	9.9%

Table S5: Age and sex breakdown of 105,867 Barts Health Emergency Hospital admissions residing within Tower Hamlets, Newham or Waltham Forest during 2013-18 inclusive.

**Barts Health Pre-COVID n = 105,867**	**White**	**Black**	**Asian**	**Mixed**	**Other**	**Unknown**
Age 16 to 19	1161	617	1529	191	212	247
Age 20 to 24	2768	925	2341	223	514	560
Age 25 to 29	3846	973	3080	238	713	779
Age 30 to 34	3706	1039	3375	195	681	814
Age 35 to 39	3265	961	3243	151	565	672
Age 40 to 44	2842	978	2709	126	502	553
Age 45 to 49	3069	1218	2196	119	473	526
Age 50 to 54	3119	1320	1932	103	450	509
Age 55 to 59	3155	1172	1959	87	392	444
Age 60 to 64	2930	698	1917	61	355	347
Age 65 to 69	3313	589	1385	44	279	288
Age 70 to 74	3217	620	1322	39	232	288
Age 75 to 79	3152	826	1673	54	224	271
Age 80 to 84	3255	741	1148	32	180	245
Age 85 and over	4742	542	751	32	217	321
Total	47540	13219	30560	1695	5989	6864
% Total	44.9%	12.5%	28.9%	1.6%	5.7%	6.5%

Table S6: Age and sex breakdown by ethnicity in the Tower Hamlets, Newham or Waltham Forest population based on the 2011 UK census.

**Tower Hamlet, Newham & Waltham Forest Census N=664295**	**White**	**Black**	**Asian**	**Mixed**	**Other**
Age 16 to 19	12068	7400	16300	2745	1957
Age 20 to 24	32548	10103	31710	4036	5611
Age 25 to 29	47264	10798	37870	3791	7379
Age 30 to 34	39888	9823	31114	2808	5458
Age 35 to 39	28682	9080	22654	2089	3631
Age 40 to 44	24936	11039	16200	1757	3211
Age 45 to 49	21841	10002	11313	1293	2148
Age 50 to 54	19002	7026	10316	946	1498
Age 55 to 59	15857	4003	8400	549	1122
Age 60 to 64	15047	2656	5262	362	852
Age 65 to 69	10637	2369	3602	278	477
Age 70 to 74	8521	2540	3965	231	423
Age 75 to 79	7560	1691	2542	159	274
Age 80 to 84	6336	894	1319	99	162
Age 85 and over	6378	483	615	67	107
Total	296565	89907	203182	21210	34310
% Total	46.0%	13.9%	31.5%	3.3%	5.3%

Table S7: *ETHICAL* diversity and absolute numbers of ethnic minority patients in comparison to other large UK reports.

	**OpenSafely First Wave**		**OpenSafely Second wave (1/9/20 to 31/12/20)**				**SGSS/CHESS (PHE report)**		**ISARIC (SSRN)**		**ONS 2/3-28/7/21**		**ETHICAL-1**		**ETHICAL-2**	
COVID-related Outcome	Death		Hospital Admission		Death		Hospital Admission		Hospital Admission		Death		Hospital Admission		Hospital Admission	
White	7119	65.5%	12120	64.3%	4874	66.2%	5149	79.6%	25547	83.2%	41855	89.6%	703	35.2%	1805	32.6%
Black	250	2.3%	362	1.9%	75	1.0%	323	5.0%	1094	3.6%	1510	3.2%	340	17.0%	634	11.5%
South Asian	608	5.6%	2100	11.1%	532	7.2%	581	9.0%	1388	4.5%	2024	4.3%	538	27.0%	1983	35.8%
Other	110	1.0%	354	1.9%	61	0.8%	414	6.4%	2664	8.7%	1307	2.8%	156	7.8%	433	7.8%
Unknown	2777	25.6%	3919	20.8%	1824	24.8%	-	-	-	-	-	-	259	13.0%	678	12.3%
Total	10864	100%	18855	100%	7366	100%	6467	100%	30693	100%	46696	100%	1996	100%	5533	100%

Table S8: Age, sex, and mortality by detailed ethnicity breakdown.

	African, n = 262[1]	Any other Asian background, n = 379[1]	Any other Black background, n = 133[1]	Any other ethnic group, n = 358[1]	Any other mixed background, n = 22[1]	Any other White background, n = 507[1]	Bangladeshi, n = 926[1]	British, n = 1,269[1]	Caribbean, n = 239[1]	Chinese, n = 30[1]	Indian, n = 301[1]	Irish, n = 29[1]	Not known, n = 509[1]	Not stated, n = 169[1]	Pakistani, n = 377[1]	White and Asian, n = 4[1]	White and Black African, n = 13[1]	White and Black Caribbean, n = 6[1]
Age[1]	55 (43, 65)	56 (43, 70)	58 (44, 68)	58 (45, 70)	53 (36, 66)	52 (39, 68)	54 (40, 68)	74 (61, 83)	72 (58, 81)	62 (44, 73)	66 (52, 77)	74 (62, 83)	52 (39, 67)	63 (49, 74)	60 (42, 71)	51 (48, 56)	57 (33, 61)	71 (53, 83)
Male	130 (50%)	225 (59%)	66 (50%)	206 (58%)	10 (45%)	273 (54%)	484 (52%)	655 (52%)	109 (46%)	11 (37%)	165 (55%)	18 (62%)	284 (56%)	91 (54%)	206 (55%)	3 (75%)	5 (38%)	3 (50%)
30-day Mortality	27 (10%)	54 (14%)	19 (14%)	44 (12%)	2 (9.1%)	59 (12%)	166 (18%)	305 (24%)	57 (24%)	4 (13%)	76 (25%)	6 (21%)	62 (12%)	30 (18%)	59 (16%)	1 (25%)	0 (0%)	3 (50%)

[1]Median (IQR); n (%)

[2]Kruskal-Wallis rank sum test

Fig S1: Patients considered in second wave *ETHICAL* study 1/9/20 to 17/2/21. Criteria: aged ≥16 first emergency admission to one of the four Barts Health acute hospitals with a positive hospital COVID-19 PCR test during or in the two weeks prior to admission. NUH: Newham University Hospital.

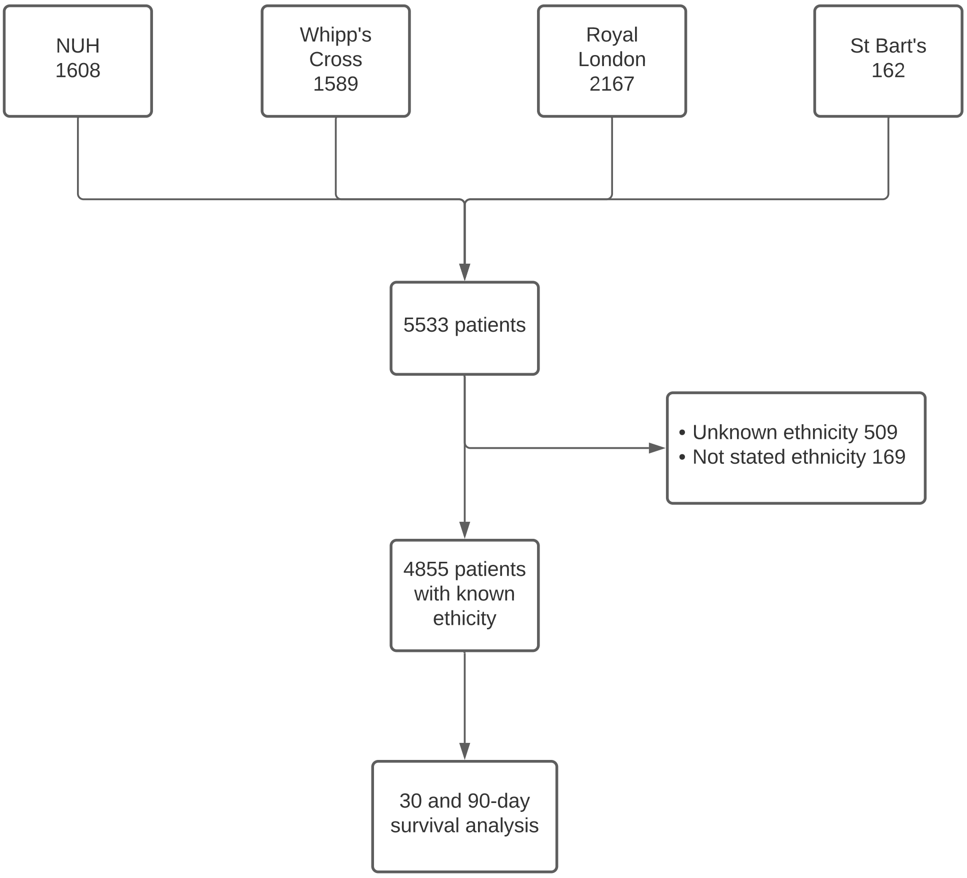

Fig S2: Daily COVID-19 associated emergency admission during our study period.

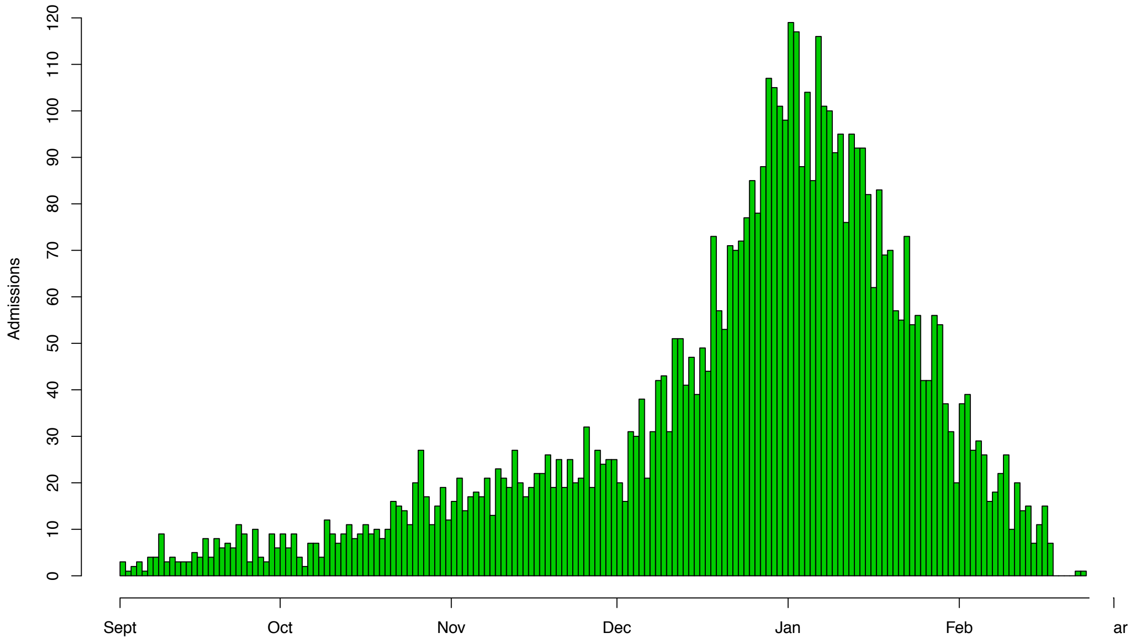

Fig S3: Cox-proportional Hazard analysis included covariates: Age, Sex, Charlson Index, Smoking history, Obesity, IMD (quintiles of local study population) for survival over 30 days in the second wave *ETHICAL* cohort.

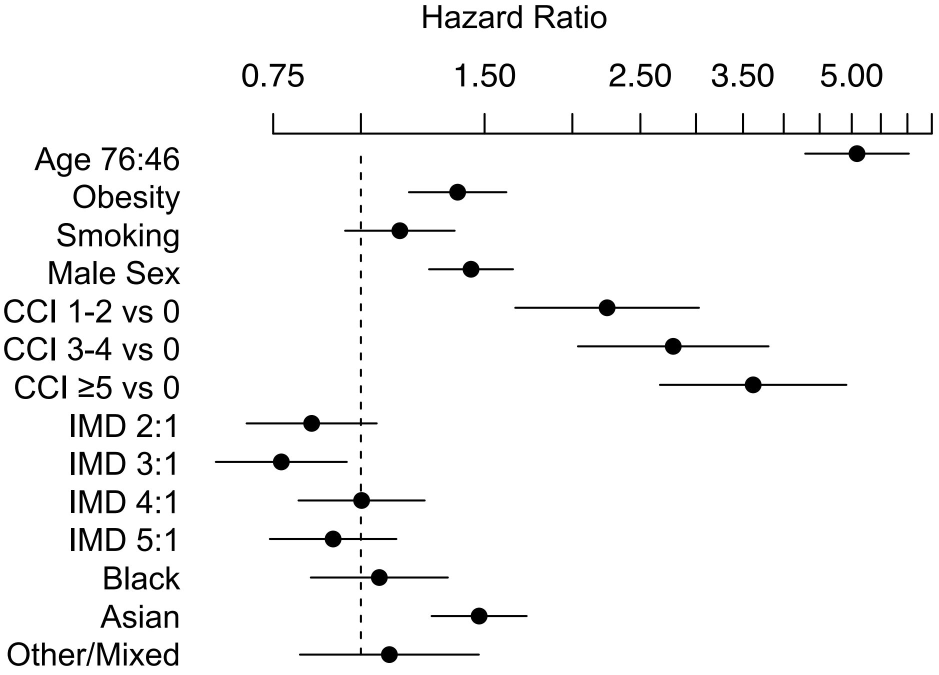

Fig S4: Cox-proportional Hazard analysis included covariates: Age, Sex, Hospital Frailty Risk Score, Smoking history, Obesity, IMD (quintiles of local study population) for survival over 30 days in the second wave *ETHICAL* cohort.

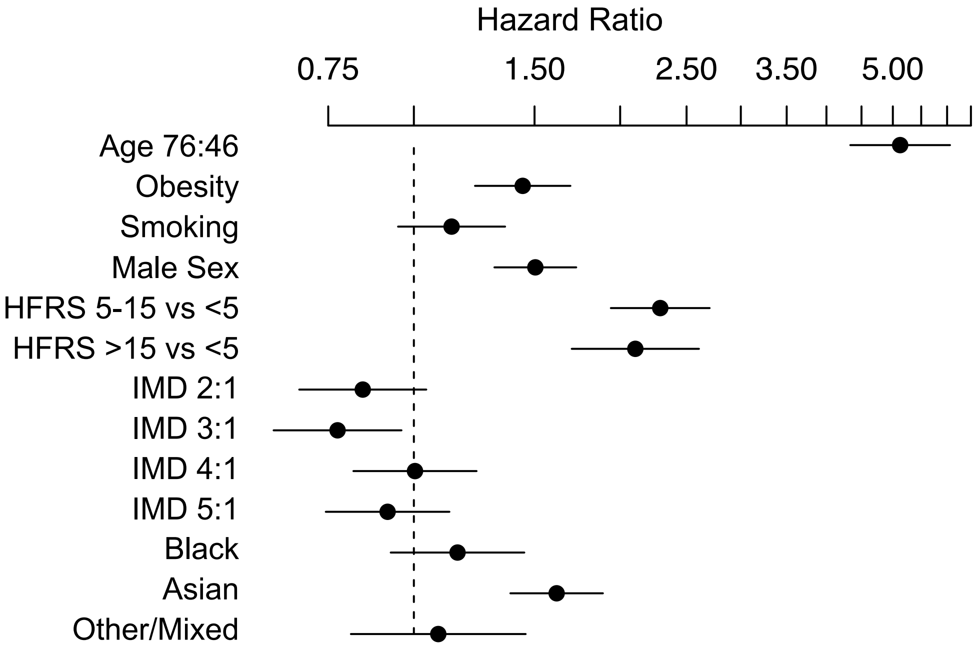

Fig S5: Logistic regression model for risk of ICU admission during first COVID associated hospitalisation in second wave adjusted for COVID-19 risk factor comorbidities.

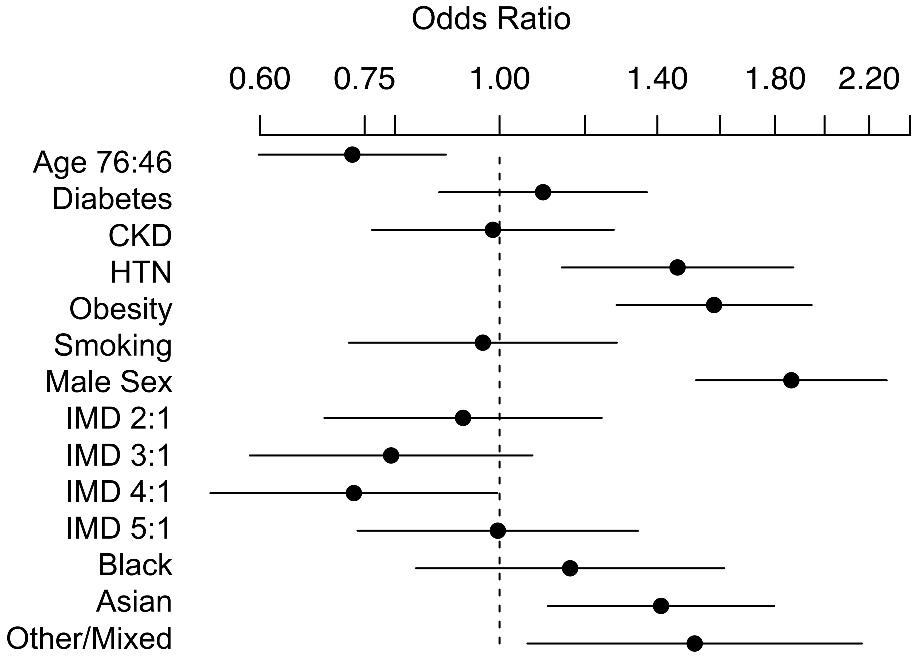

Fig S6: Cox-Hazard analysis (unstratified) for survival of first wave patients to 12 months.

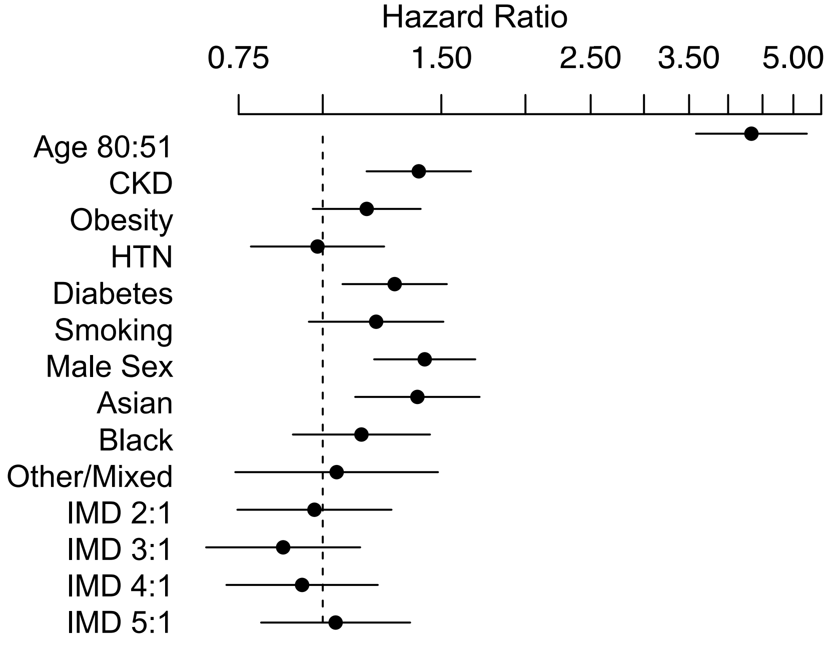

Supplement: Supplementary file 1 — Supplementary Information. [file 41598_2022_7532_MOESM1_ESM.pdf]
